# Effects of sumac aqueous extract along with eating and drinking modification on functional dyspepsia symptoms in comparison with omeprazole: An open-label, randomized, controlled clinical trial

**DOI:** 10.22038/ajp.2024.25239

**Published:** 2025

**Authors:** Mahdi Saravani, Zahra Memariani, Nasser Behnampour, Mohammadreza Seyyedmajidi, Fatemeh Kolangi

**Affiliations:** 1 *Golestan Research Center of Gastroenterology and Hepatology, Department of Persian Medicine, School of* *Persian Medicine, Golestan University of Medical Sciences, Gorgan, Iran.*; 2 *Pharmaceutical Sciences Research Center, Babol University of Medical Sciences, Babol, Iran. *; 3 *Department of Persian Medicine, School of Persian Medicine, Babol University of Medical Sciences, Babol, Iran.*; 4 *Department of Biostatistics, Faculty of Health,Golestan University of Medical Sciences,Gorgan, Iran*; 5 *Golestan Research Center of Gastroenterology and Hepatology, Golestan University of Medical Sciences, Gorgan, Iran*; 6 *Counseling and Reproductive Health Research Centre, Department of Persian Medicine, School of Persian Medicine, Golestan University of Medical Sciences, Gorgan, Iran.*

**Keywords:** Functional dyspepsia, Persian medicine, Sumac, Rhus coriaria L., Quality of life

## Abstract

**Objective:**

Functional dyspepsia (FD), a common gastrointestinal problem. The aim of the study was to evaluate the influence of *Rhus coriaria* L. (sumac) on FD.

**Materials and Methods:**

This randomized controlled clinical trial study included 104 patients aged 18 to 60 years diagnosed with FD according to the ROME IV criteria. Four groups were formed: A) sumac extract + dietary changes, B) dietary changes, C) sumac extract and D) omeprazole. During the present eight-week study, patients' FD symptoms were assessed using the Gastrointestinal Symptom Rating Scale (GSRS) in four sessions. The Nepean Dyspepsia Index (NDI-10) was used to measure the impact of the interventions on patients' quality of life.

**Results:**

The study employed generalized estimating equation (GEE) analysis and found that symptom severity decreased across all groups during the intervention period. At the fourth week, no notable difference was noted between the omeprazole group and others. After the intervention, the severity of symptoms increased, especially in the omeprazole group, resulting in a significant difference compared to other groups.

**Conclusion:**

It seems that as a complementary treatment in FD, sumac might be effective with a more lasting effect with a significantly less recurrence of symptoms.

## Introduction

Dyspepsia is one of the most common gastrointestinal disorders (Harmon and Peura 2010). According to Rome IV criteria, dyspepsia is characterized by heartburn, epigastric pain, early satiety, and postprandial fullness. Dyspepsia can also refer to symptoms such as feeling full quickly, nausea, frequent vomiting, loss of appetite, and frequent belching (Stanghellini et al. 2016).

From an etiological point of view, dyspepsia is divided into two subgroups: secondary and functional. In the secondary type, which affects 40% of patients with dyspepsia, there is an organic cause that justifies the symptoms (Aghazadeh et al. 2005; Jones 2002). Functional dyspepsia (FD) refers to a combination of the following symptoms such as postprandial fullness, early satiation, epigastric pain, and epigastric burning (Stanghellini et al. 2016). 

The global prevalence of dyspepsia varies from 1.8 to 57.0%. This diversity can be attributed to the country and the criteria used to define dyspepsia (Ford et al. 2015). Some factors such as dietary habits, social and cultural differences, psychological issues, and gastrointestinal (GI) infections influence dyspeptic symptoms, resulting in variation in the distribution of prevalence(Ghoshal et al. 2011). Studies have shown that the prevalence of dyspepsia varies in different cities of Iran due to differences in lifestyles, cultural backgrounds, dietary habits, etc.(Amini et al. 2012). About 10 to 45% of people complain of this condition during their lifetime, of which only one in 4 patients refer to a doctor, and 25% of these patients go for further evaluation such as endoscopy and ultrasonography in Western population (Arents et al. 2002). Based on previous studies conducted in Iran, the prevalence of dyspepsia has been reported between 8.5 to 29.9% of the general population (Aghazadeh et al. 2005; Barzkar et al. 2009; Khademolhosseini et al. 2010).

The pathophysiology of FD is poorly understood, so, some drug treatment recommendations control patients' symptoms(Eftekharafzali et al. 2018). Unfortunately, common medicines have limited therapeutic efficacy, Unfortunately, common medicines have limited therapeutic efficacy, and over 50% of patients with FD request alternative treatments(Kim et al. 2017). 

Various treatments are available for FD, such as proton pump inhibitors and H2 blockers, prokinetics, antidepressants, acotiamide, and vonoprazan(Yamawaki et al. 2018). 

Long-term use of these drugs has both advantages and disadvantages, especially when infections and bacterial growth occur, leading to diseases such as atrophic gastritis, malabsorption, acute intestinal nephritis, and symptoms such as diarrhea, constipation, drowsiness, and muscle pain (McColl 2009; Sampathkumar et al. 2013; Yibirin et al. 2021).

Therefore, other treatment methods such as complementary and alternative medicines such as herbal medicine are also suggested for gastrointestinal diseases (Anheyer et al. 2017; Langmead and Rampton 2001). The usefulness of some herbal drugs has been shown in functional dyspepsia like Menthacarin (a combination of peppermint and caraway oil) or Rikkunshito (a blend of 8 different herbs)(Rich et al. 2017b; Yamawaki et al. 2018). Sumac (*Rhus coriaria* L.) is a herb famous in the Mediterranean regimen(Khalil et al. 2021b). Sumac (*Rhus coriaria* L.) is a plant in the family Anacardiaceae which includes about 81 genus and over 800 species (Ghahreman and Okhovvat 2010). The aqueous extract of sumac fruit and its ethanolic extract contain compounds such as phenolic acids, flavanol, and gallic acid, which have antioxidant effects (Isik et al. 2019). Recent studies have shown that the flavonoids and tannins of sumac in its hydroalcoholic extract have a protective effect against ulcers and reduce the ulcer index in rat model, which could be used in the treatment of gastrointestinal problems (Ahmad et al. 2013). Furthermore, in isolated rabbit jejunum, *R.* *coriaria* was shown to exhibit concentration-dependent anticonvulsant effects, possibly via the Ca++ antagonist pathway, providing evidence for its use in abdominal cramps(Janbaz et al. 2014). 

 In traditional Persian medicine books, sumac has many properties, including the effects of strengthening the stomach and digestion(Shirbeigi et al. 2015). The beneficial effect of sumac is recognized in various conditions like non-alcoholic fatty liver disease, necrotizing enterocolitis, and diabetes mellitus, and in controlling abnormal bleeding based on Persian medicine(Isik et al. 2019; Kazemi et al. 2020; Zakeri et al. 2020). However, there have been no randomized clinical trials conducted to evaluate the effects of sumac on FD or to compare its effects with standard medications, such as omeprazole. Therefore, this study aimed to compare the efficacies of sumac aqueous extract on FD symptoms in comparison with omeprazole.

## Materials and Methods

### Trial design

The present study is a randomized controlled clinical trial (RCCT) conducted from March to August 2021. Patients with one of the complaints of dyspepsia who were referred to Dr. Mousavi Hospital (Gorgan, Golestan province, Iran), were invited to participate in this study.

The Ethics Committee approved this trial at the Golestan University of Medical Sciences (Ethics committee reference number: IR.GOMS.REC.1399.298). The trial was also registered in the Iranian Clinical Trials Registry (IRCT) (ID number: IRCT20200424047192N1). Written consents were obtained from all of participants of this study. 

### Participants

We performed endoscopy for all patients to rule out pathologic findings. In addition, we assessed Helicobacter pylori (*H. pylori*) infection with serologic markers to determine whether *H. pylori* infection was present. Patients without pathologic findings in endoscopy procedure and no *H. pylori* infection were evaluated using the ROME III standard Persian questionnaire in terms of suffering from FD. If they had the diagnostic criteria of this questionnaire, they were included in the study.

### Inclusion criteria

We included patients with the following features: Age between 18 and 60 years; the presence of at least one of the symptoms of epigastric pain, heartburn, postprandial fullness, and early satiety; and onset of symptoms at least from 6 months ago and presence of symptoms at least three days a week within the last three months. 

### Exclusion criteria

Any pathological findings in the endoscopy procedure; *Helicobacter pylori* infection; use of antibiotics or PPIs during the previous month; any underlying diseases including heart failure, renal or hepatic failure, cirrhosis, diabetes, or thyroid disorders; smoking, drugs or alcohol consumption; consumption of non-steroidal anti-inflammatory drugs (NSAIDs) or aspirin; pregnancy or breastfeeding.

### Interventions

This study was designed as a two-phase trial in four parallel treatment groups, and participants ‎were evaluated four times during the study period. The first phase of the study was the intervention ‎phase when the patients in the medication groups received treatment for four weeks. In the second ‎phase, patients stopped medication and were followed up for four more weeks. Participants were ‎evaluated before the initiation of the study, and in the second, fourth and eighth week. One hundred and ‎four patients were assigned to the following four groups based on Permuted-block randomization ‎method. Group A patients received education on modification of eating and drinking habits and ‎sumac extract capsules, group B patients received education on modification of eating and drinking ‎habits, group C patients received sumac extract capsules, and group D patients received ‎omeprazole capsules.‎

In the intervention phase, at the first visit (day 1), group A and B patients received a specific and ‎educational package including written instruction sheets as well as oral explanations by a trainer ‎regarding proper behavior of eating and drinking. In addition, group A and C patients received 84 ‎capsules of sumac extract along with a medication guide sheet on the first visit (day 1), which was ‎taken 6 capsules per day, two after each meal. At the second visit, which was done two weeks after ‎the start of the study, 84 capsules of sumac extract were delivered to them in the same way.‎

Group D patients received 14 capsules of 20 mg of omeprazole on the first visit (day 1), and one ‎capsule was taken every day before breakfast. At the second visit, which was done two weeks after ‎the start of the study, 14 capsules of 20 mg omeprazole were delivered to them for consumption in ‎the same way.‎

### Drug preparing process

Dried sumac fruits from Iran's Kurdistan Province were purchased from a professional medicinal herb seller and authenticated by a botanist (Dr. Tayebeh Amini). Voucher specimens (*Rhus coriaria* L. No. 11529 HNBG) were deposited in the herbarium of the Nowshahr Botanical Garden.

To prepare the extract, first, the fruits of the brown sumac plant were cleaned. Then, the kernels were separated from the mesocarp and only the powder of the sumac mesocarp was used to make medicine. 

A certain amount of powder was boiled in a closed container with a ratio of 1 to 6-8 (w/w) in distilled water for 20 min with gentle heat. After cooling, the contents of the container were strained and the obtained extract was concentrated by heat bath at a temperature of 45-50°C until reaching a constant weight. The extraction yield was 60%.

The extract was granulated by adding excipient, re-dried at room temperature, and turned into a powder. Finally, the powder with a weight equivalent to 500 mg of sumac mesocarp was filled in capsules.

After microbial limit tests, sumac extract powders were filled in gelatin capsules by a manual capsule-ﬁlling machine.

Total bacteria, molds and yeasts, absence of* Escherichia coli*, and *Salmonella* were tested in Food, Cosmetics and Hygiene Control Laboratory of Golestan University of Medical Sciences.

### Determination of total phenolic compounds

Analysis of the total phenolic compounds of the dry aqueous extract of sumac powder with a spectrophotometric method was performed in Professional Center of Analysis, Institute of Medicinal Plants, Karaj, Iran. 

### HPLC analysis of gallic acid

Sumac extract was analyzed in term of gallic acid content, by High Performance Liquid Chromatography (HPLC) analysis in Professional Center of Analysis, Institute of Medicinal Plants, Karaj, Iran. Briefly, the extract was passed through a filter (0.45 μm) before injection. HPLC column was 150 mm in length (Agilent technologies 1200 series). The mobile phase: (A) acidified water with formic acid (0.1%) and (B) acidified acetonitrile with formic acid (0.1%). Injection volume = 20 μl, analysis time: 60 min; the flow rate: 0.8 mL/min, the wavelengths (UV 300 nm), temperature: 30°C. Reference substance was gallic acid.

### Study outcomes

Total score of gastrointestinal symptom rating scale, Description of Quality of Life, and Eating and drinking habits were the primary outcomes of this study. These items were measure using Gastrointestinal Symptom Rating Scale (GSRS) questionnaire, 10‐item short form of the Nepean Dyspepsia Index (NDI-10), and Eating and drinking habits questionnaire which were used at baseline, and 2, 4, and 8 weeks after starting the intervention.

 This trial’s secondary outcomes evaluated the occurrence of adverse effects in the studied groups.

The questionnaire related to demographic characteristics, was completed at the first visit of patients (day 1). In addition, the Gastrointestinal Symptom Rating Scale (GSRS) was completed for the individuals at all visits. This scale is a disease assessment tool based on the examination of gastrointestinal symptoms and clinical experience, and is used to evaluate common symptoms of the gastrointestinal diseases. This tool consists of 15 questions, and each question is graded on a 7-point Likert scale from discomfort (point 1) to extreme discomfort (point 7). This questionnaire has been approved for functional gastrointestinal disorders in other researches with 4 dimensions: abdominal pain, indigestion, constipation, and diarrhea. The total score comes from the sum of the average scores in each subscale and shows the severity of symptoms. In the present study, validated Persian version of GSRS questionnaire was used(Mazaheri and SadatKhoshouei 2012). Also, we adjusted the standard GSRS questionnaire to assess postprandial discomfort dimension of FD by adding two questions about early satiation and postprandial fullness. In this study, we also assessed the impact of symptoms on the quality of life of the patients. The validated Persian version of short form Nepean dyspepsia index (NDI-10) was used for this regard in all the patients visits(Azimi et al. 2017). With this questionnaire which includes 5 dimensions, the impact of symptoms on the quality of life of patients with FD is evaluated by a total of 10 questions. In model one analysis, the total score of adjusted GSRS score and without considering any covariates, only the group C had a significant difference with omeprazole in this study. In model 2 analysis, the total adjusted GSRS score with considering first visit score as a covariate, was significantly different in group A and C compared to omeprazole group. In model three analysis, we considered age and Body mass index (BMI)and first visit as covariates.

### Recording possible adverse effects of the medications

 During treatment, the drugs were delivered to the participants every 2 weeks. Still, until the end of 4 weeks after the end of drug treatment they were checked for medicine consumption and possible unwanted side effects of the drug.

### Sample size

The sample size was calculated using the mean and standard deviation based on Xiong et al study(Xiong et al. 2019) at a confidence level of 0.95 and a power of test of 0.90, ${\mathrm{sd}}_{1}=5.6$, ${\mathrm{sd}}_{2}=5.1$, $\mu_{1}=22.5$ and $\mu_{2}=28.6$ for each group was determined equal 15. Considering the number of four groups and applying the formula$n^{*}=\sqrt[{2}]{K-1}\times n$ , the sample size in each group was determined to be 26. Thus, based on the random allocation, eligible patients were assigned to four treatment groups. Due to the type of the interventions, blinding was not possible in this study.

### Randomization and allocation ‎concealment

In this study, 104 eligible patients were assigned to four groups according to the block randomization method (the size of each block was 8, such as ABBCADCD). The statistical consultant, who was not involved in the selection of patients and their assignment to groups, created the random sequence. The gastroenterology resident enrolled participants, and the graduate student assigned interventions to participants. Among all possible states of blocks of size 8, 13 blocks were randomly selected using a uniform distribution in R software.

### Statistical analyses

Data were analyzed by SPSS software version 21. Mean and standard deviation were used to describe continuous variables.

The normality of the data was checked using the Shapiro-Wilk test and the homogeneity of variances was checked using the Levene test. Analysis of variance was used for normal data and Friedman and Kruskal-Wallis tests were used for Abnormal distributed.

The generalized structural equation method (GEE) was used to determine the effect of interventions on the response variable in the presence of other intervening variables.

## Results

### Demographic data

**Figure 1:**
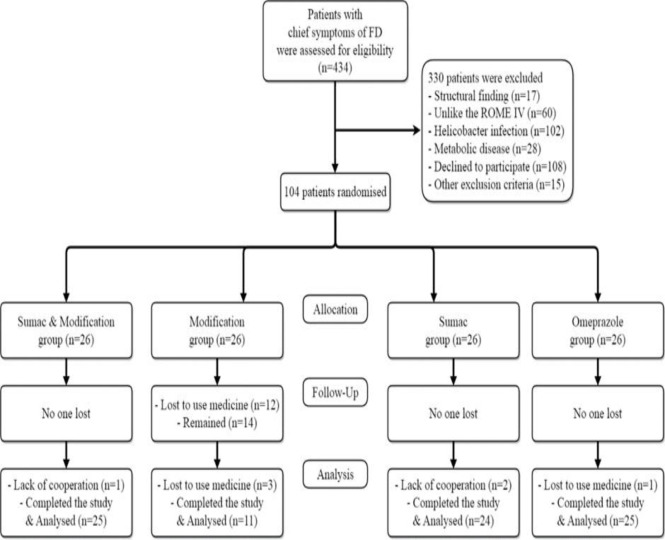
CONSORT diagram of study population

 In the present study, 434 participants were screened and 303 of them were excluded due to not meeting the inclusion criteria. Then, 104 patients diagnosed with FD were enrolled. During the study period, information of 19 patients was lost and finally, information of 85 patients was analyzed. The CONSORT flow chart of patients entering the study is shown in Figure 1.

The average age of the study participants was 42.8±11.3 years old. Also, 65.4% of the participants were female, and 53.8% had less than 12 years of education. Based on the results, the patients' average age, body mass index, abdominal circumference, and literacy level showed no significant difference among the groups. The demographic information of patients is shown in Table 1. The frequency distribution of gender, mean age and baseline values in patients who were lost to follow-up were compared with those who completed the study. There was no statistically significant difference between the two groups in any of these traits.

### Assessments of outcomes

#### Total adjusted GSRS score (15+2 questions)

The results of analysis showed significant difference between group A and C and omeprazole group in the Fourth visit. The effect of Body Mass Index** (**BMI) and age on the results was insignificant in model three analysis, hence we did not considered them covariates in the models four, five and six analyses.

 In the model four analysis with considering first visit as a covariate, the total adjusted GSRS score was significantly different between the group B and omeprazole group in the second visit. In model five analysis with considering first visit as covariate, the total adjusted GSRS score was not significantly different between group A and B and C and the omeprazole group in the third visit. In model six analysis with considering first visit as covariate, the total adjusted GSRS score was significantly different between group A and B and C and the omeprazole group. The result of GEE analysis on total adjusted GSRS score is shown in Table 2. 

#### Total standard GSRS score

In model one analysis, the total GSRS score without considering any covariates was significantly different between group C and omeprazole group. In model two analysis with considering first visit as covariate, there was a significant difference between groups A and C with omeprazole group. In the third model with considering first visit, age and BMI as covariates, significant difference was observed between group A and C with the omeprazole group. The effect of age and BMI was insignificant so we did not consider them covariates in the following analysis models. In model four analysis with considering first visit as covariate, there was a significant relationship between group B and omeprazole group in the second visit. In model five analysis with considering first visit as a covariate, no significant difference was observed within the groups in the third visit. In model six analysis with considering first visit as covariate, the total GSRS score was significantly different between group A, B and C and the omeprazole group in the fourth visit. The result of GEE analysis on total GSRS score is shown in Table 3. 

#### Total quality of life score based on NDI-10 scale

In model one analysis, the impact of symptoms on quality of life was assessed without considering any covariates. A significant difference was observed between group A and omeprazole group. In model two analysis considering first visit as a covariate, a significant relationship between group A and omeprazole group was observed. In the model three analysis considering first visit, age and BMI as covariates, significant difference was observed between group A and omeprazole group. However, the effect of age and BMI was not significant hence we did not consider them as covariates in the following analysis models. In model four analysis considering first visit as covariate, group B showed significant difference compared to omeprazole group in the second visit. In the model five analysis considering first visit as covariate, no significant difference was observed between groups in the third visit. In model four analysis considering first visit as covariate, significant difference was observed between group A and C with omeprazole group in the fourth visit. The result of GEE analysis on impact of symptoms on quality of life score based on NDI-10 scale is shown in Table 4**.**

### Primary outcomes

**Figure 2:**
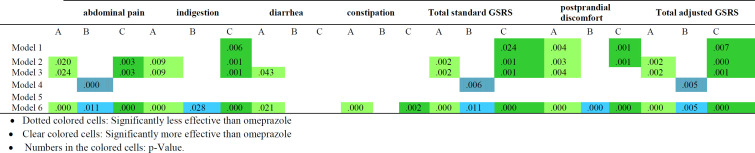
Severity of symptoms by time

**Figure 3:**
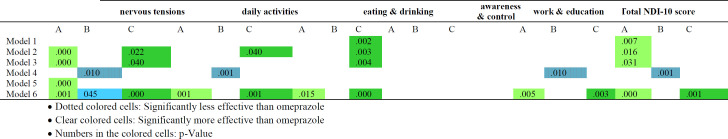
Impact of symptoms on quality of life by time

Based on the results of our study, symptoms in all groups decreased with a significant slope at the beginning of the intervention until the second week, and this decrease continued with a lower slope to the fourth week. At the fourth week, no notable difference was noted between the omeprazole group and others. By stopping the intervention in the fourth week, the severity of symptoms in all groups started to increase, but the steep slope of this increase in the omeprazole group caused a significant difference compared to the other groups, which indicates that the return of symptoms in participants in the omeprazole group was significantly higher than in that of other groups (Table 5 and Figure 2). As shown in Figure 3, the impact of symptoms on quality of life in different visits also has a similar trend to the severity of symptoms in groups (Figure 3).

The results regarding the dimensions related to the co-occurring symptoms of dyspepsia show that in the diarrhea dimension, only group A had a significant difference in symptom improvement compared to the omeprazole group in Models 3 and 6. In the dimension of constipation, there is only a significant difference between the A and C groups compared to the omeprazole group in model 6. The most significant results are observed in the dimensions related to the main symptoms of FD, in groups A and C and in model 6.

Significant differences between omeprazole and other interventions in subscores and total scores of GSRS are shown in Table 6.

The results regarding dimensions of impact of symptoms on quality of life show that there is not any significant difference in the awareness and control dimension. The most significant results belong to the nervous tensions dimension. Significant differences between omeprazole and other interventions in subscores and total scores of NDI-10 are shown in Table 7.

### Secondary outcomes

There were no complications or adverse events during the study.

### Chemical analysis of the preparation

The amount of total phenolic extract powder was 75.08 to 5.90 mg gallic acid equivalent/g and for gallic acid 12.5 mg/g (supplementary data). The permissible range of microbial contamination corresponded to the pharmacopoeia.

## Discussion

Functional dyspepsia is a common gastrointestinal disorder with symptoms such as epigastric pain, painful burning, early satiety, and postprandial fullness (Talley et al. 2017). This disease affects the quality of life of the individual(Aro et al. 2011; Lacy et al. 2013). There is no specific treatment for this disease.. Some studies suggest anti-acid and prokinetic therapies(Yamawaki et al. 2018). Management of dyspepsia is challenging because of the multifactorial nature and heterogeneity of symptoms(Ford et al. 2015); more, the available treatment options are only moderately effective(Moayyedi et al. 2017). Traditional medicine and herbal remedies are popular treatment options, especially in Asia(Ekor 2014; Ernst 2001; Lazarou and Heinrich 2019). Previous studies suggest the promising effect of herbal medicine in different diseases, especially gastrointestinal diseases(Kim et al. 2020). Different studies have shown that medicinal plants can be effective on dyspepsia via several mechanisms. Herbal products often contain compounds with multiple reported pharmacological effects on gastrointestinal motility and secretory functions, as well as cytoprotective and psychotropic properties (Gwee et al. 2021).

This RCCT study was conducted with the aim of comparing the effect of sumac capsules as a traditional herbal medicine with omeprazole in improving the symptoms of people with FD. 

Sumac (*Rhus* spp.) contains a plethora of bioactive compounds, including polyphenols, flavonoids, tannins, and organic acids. These constituents exhibit various pharmacological properties such as antioxidant, anti-inflammatory, antimicrobial, and gastroprotective effects. The synergistic action of these compounds might possibly contribute to the overall therapeutic efficacy of sumac (Zhou et al. 2020). 

Based on the results of this study, FD patients using omeprazole showed a reduction in their symptoms. Previous studies also suggest the effectiveness of omeprazole in treating FD patients compared to other treatments like H2 antagonists (Goves et al. 1998; Mason et al. 1998; Moayyedi et al. 2017; Sakurai et al. 2012). Moreover, sumac as a herbal medicine also was effective in healing symptoms of FD. This finding was consistent with other studies evaluating herbal therapy in FD. In a meta-analysis study by Ko et al., a herb called Rikkunshito effectively improved FD (Ko et al. 2021). In another meta-analysis by Heiran et al., the effect of herbal medicine on FD compared to placebo was reported (Heiran et al. 2022). 

In our study, no significant difference was observed between the omeprazole consuming group and other groups during the intervention period, except for group B, which had a significantly higher GSRS total score compared to omeprazole in the second week. Therefore, the difference in the effectiveness of these interventions in improving FD was generally not significant during the intervention period. This finding is in contrast with the study of Bordbar et al. which used herbal therapy by a combination of *Trachyspermum ammi* L., *Anethum graveolens* L., and *Zataria multiflora* Boiss oils and showed a significant difference in reducing FD symptoms compared to omeprazole after four weeks(Bordbar et al. 2020). 

Although no significant difference was observed between omeprazole and other groups after four weeks of the treatment period, four weeks after the end of the intervention, significant changes in persistent effect were observed. The GSRS total score was significantly higher in the omeprazole group compared to the other groups. These findings showed a more persistent effect of sumac and habitual modification on FD symptoms, either together or each alone, compared to omeprazole. More, it seems that the recurrence of FD symptoms is another important factor that needs to be discussed. In one study by Reimer et al., (Reimer and Bytzer 2010), it has been shown that short-term PPI therapy was superior to placebo in patients with symptoms recurrence. While, in their other trial, PPI therapy for longer period of time (8 weeks) induced acid-related symptoms in participants after withdrawal (Reimer et al. 2009). Which might be caused by the rebound acid hypersecretion.

Since FD is a multifactorial disease, the effect of herbal medicines on gastrointestinal symptoms may have different aspects. Various studies have shown that medicinal plants can be effective in improving symptoms through various mechanisms affecting the movement of the gastrointestinal tract and secretions of the digestive system, cellular protection, and psychological effects (Gwee et al. 2021). Sumac (*Rhus coriaria* L.) is one of the famous herbs, especially in the Mediterranean regimen(Tohma et al. 2019). Sumac has been traditionally used to treat gastrointestinal diseases such as diarrhea and gastric ulcers (Sakhr and El Khatib 2020). There is evidence that sumac can have different effects on humans and animals. In addition to its antioxidant properties, sumac has antibacterial and neuroprotective properties (Akbari-Fakhrabadi et al. 2018; Alsamri et al. 2021; Khalil et al. 2021a; Nasar-Abbas and Halkman 2004; Sakhr and El Khatib 2020). More, it has been shown that *R. coriaria* possesses antispasmodic effects in isolated rabbit jejunum, possibly via Ca++ antagonist pathway(Janbaz et al. 2014). Previous studies showed the role of inflammation in FD (Walker and Talley, 2017). The anti-inflammatory effect of sumac has been demonstrated in some studies(Akbari-Fakhrabadi et al. 2018; Alsamri et al. 2021; Sakhr and El Khatib 2020). Ahmad et al. and Haqeeq et al showed the anti-ulcer effect of a sumac extract (145 and 248 mg/kg) in animal models of stress-, ethanol-, and indomethacin-induced gastritis (Ahmad et al. 2013; Haqeeq et al. 2015). This effect of sumac is due to its phenolic content and is considered to be related to nuclear factor kappa B (NF-kB), as the main anti-inflammatory mechanism (Martinelli et al. 2022). More, as its one of the main chemicals, gallic acid has been shown with gastroprotective effects on ethanol-induced gastric ulcer in rats via Nrf2/HO-1 anti-oxidative pathway (Zhou et al. 2020). 

There is no previous clinical trial that evaluated sumac efficacy in FD. But similar to our study, Bordbar *et al*. evaluated the efficacy of a herbal preparation containing *Zataria multiflora,* *Trachyspermum ammi*, and *Anethum graveolens*, in FD patients by using GSRS as measurement tool prescribed omeprazole as control; similar to our results, a significant decrease was observed in total GSRS scores, and also the quality of life was improved at the end of the intervention (Bordbar et al. 2020). 

Based on the results of our study, except for group B, which had a significantly higher NDI-10 total score compared to omeprazole in the second week, no significant difference was observed between the group that consumed omeprazole and other groups during the intervention period. Therefore, the difference in the effectiveness of these interventions in reducing the impact of symptoms on the quality of life of FD patients was generally not significant during the intervention period. Although no significant difference was observed between omeprazole and other groups after four weeks of the treatment period, significant changes were observed four weeks after the end of the intervention. The NDI-10 total score was significantly higher in the omeprazole group compared to the sumac user groups. These results demonstrated a more sustained effect of sumac and habit modifications on improving quality of life, either together or sumac alone, compared to omeprazole in FD patients.

The quality and manner of eating a meal are essential factors in the occurrence of symptoms in people with FD. Patients often seek dietary advice that can alleviate their symptoms. The type of diet, eating behaviors, irregular eating patterns and also the speed of eating significantly affect the symptoms of functional indigestion and patients’ quality of life(Duboc et al. 2020). 

Several studies that have investigated the effect of various herbal preparations on the symptoms of functional dyspepsia have shown the effect of these herbs on the quality of life of FD patients. Artichoke (*Cynara scolymus*) is a herb with promising effects on quality of life of FD patients(Holtmann et al. 2003).

 Menthacarin is another herb with effective properties in quality of life of FD patients(Rich et al. 2017a).

This study has some limitations. First, due to the nature of the procedure, blinding could not be performed. Only one clinical center was involved in our study. The patients were only followed for four weeks. Although sumac has been shown to be effective, long-term effectiveness and safety could not be determined. Nevertheless, this study is the first randomized controlled trial to confirm the effectiveness of sumac in treating FD and improving quality of life compared to any of the widely used therapies. We recommend further research into the optimal dosage and duration of sumac in FD. Even after some evidence indicating the potential role of sumac in improving *H. pylori*-related gastritis (Martinelli et al. 2022) , it is better to design studies that examine the effects of sumac on FD patients with *H. pylori *gastritis

As a herbal medicine, sumac has various health-promoting properties. This plant is traditionally used to treat indigestion (Shirbeigi et al. 2015). In addition, there is evidence of anti-inflammatory, antispasmodic and antioxidant properties(Alsamri et al. 2021). Its chemical compounds, such as phenolic compounds, especially gallic acid, are also said to have protective effects on the digestive system(Isik et al. 2019). According to the results of this study, the use of sumac as a complementary treatment for FD appears to have a sufficient effect on the severity of symptoms and results in a more sustained effect, so that long-term drug use is not necessary. Further clinical studies are needed to validate these results. Additionally, elucidating the optimal dosage, duration, and formulation of sumac-based intervetntions is critical to maximizing their effectiveness and safety. In addition, investigating possible interactions with conventional medications and exploring novel delivery systems may improve the clinical utility of sumac in gastrointestinal health.

**Table 1:** Baseline characteristics of randomized patients in 4 groups

**Variable**	**Sumac and Modification group (group A)**	**Modification group (group B)**	**Sumac group (group C)**	**Omeprazole group (group D)**	**P-value**
Age (Mean ± SD)	41.54 ± 10.89	45.77 ± 11.68	40.88 ± 11.13	43.15 ± 11.50	0.293*
BMI (Mean ± SD)	28.36 ± 4.82	27.13 ± 4.07	28.32 ± 5.55	26.12 ± 3.51	0.235**
WC (Mean ± SD)	100.53 ± 3.10	96.76 ± 3.23	101.84 ± 3.75	94.15 ± 2.37	0.298 **

BMI: Body mass index WC: Waist circumference. **Kruskal-Wallis Test **ANOVA Test

**Table 2:** GEE analysis on total adjusted GSRS score

**Models**	**Parameter**	**β**	**95 % CI**	**p-value**
Model 1 (without considering any covariate)	Group D	-		
	Group A	-0.242	-0.563, 0.078	0.139
	Group B	-0.021	-0.389, 0.348	0.912
	Group C	-0.390	-0.676, -0.105	0.007
Model 2 (considering first visit a covariate)	Group D	-		
	Group A	-0.385	-0.626,-0.143	0.002
	Group B	0.024	-0.251,0.299	0.864
	Group C	-0.357	-0.554,-0.159	0.0004
	First visit Score	0.403	0.283,0.523	4.3547E-11
Model 3 (considering first visit, age and BMI covariates)	Group D	-		
	Group A	-0.386	-0.635, -0.136	0.002
	Group B	0.028	-0.255, 0.310	0.848
	Group C	-0.360	-0.563, -0.157	0.001
	First visit Score	0.401	0.279, 0.523	1.2043E-10
	Age	-0.002	-0.010, 0.006	0.638
	BMI	0.000	-0.018, 0.018	0.989
Model 4 (mean score in the second(W*2) visit with considering first visit covariate)	Group D	-		
	Group A	-0.097	-0.333, 0.138	0.417
	Group B	0.323	0.095, 0.550	0.005
	Group C	-0.132	-0.323, 0.059	0.176
	First visit score	0.454	0.346, 0.563	2.2204E-16
Model 5 (mean score in the third(W4) visit considering first visit covariate)	Group D	-		
	Group A	-0.241	-0.533, 0.051	0.106
	Group B	0.122	-0.233, 0.478	0.500
	Group C	-0.191	-0.426, 0.045	0.112
	First visit score	0.300	0.165, 0.435	1.4E-5
Model 6 ( mean score in the fourth(W8) visit with considering first visit covariate)	Group D	-		
	Group A	-0.833	-1.138, -0.527	9.4524E-8
	Group B	-0.576	-0.976, -0.177	0.005
	Group C	-0.766	-1.046, -0.486	8.206E-8
	First visit score	0.455	0.301, 0.608	6.5639E-9

**Table 3:** GEE analysis on total GSRS score

**Models**	**Parameter**	**β**	**95 % CI**	**p-value**
Model 1 (without considering any covariate)	Group D	-		
	Group A	-0.146	-0.442, 0.150	0.333
	Group B	0.045	-0.318, 0.408	0.807
	Group C	-0.301	-0.562, -0.040	0.024
Model 2 (considering first visit a covariate)	Group D	-		
	Group A	-0.349	-0.568, -0.131	0.002
	Group B	0.043	-0.211, 0.298	0.739
	Group C	-0.314	-0.492, -0.136	0.001
	First visit Score	0.419	0.310, 0.528	3.908E-14
Model 3 (considering first visit, age and BMI covariates)	Group D	-		
	Group A	-0.351	-0.575, -0.128	0.002
	Group B	0.049	-0.214, 0.313	0.713
	Group C	-0.319	-0.504, -0.133	0.001
	First visit Score	0.416	0.307, 0.525	8.8485E-14
	Age	-0.003	-0.010, 0.004	0.398
	BMI	0.000	-0.017, 0.017	0.988
Model 4 (mean score in the second (W2) visit with considering first visit covariate)	Group D	-		
	Group A	-0.083	-0.302, 0.137	0.459
	Group B	0.295	0.086, 0.504	0.006
	Group C	-0.124	-0.305, 0.057	0.181
	First visit score	0.462	0.359, 0.564	0.0E0
Model 5 (mean score in the third (W4) visit considering first visit covariate)	Group D	-		
	Group A	-0.227	-0.491, 0.038	0.093
	Group B	0.141	-0.192, 0.474	0.407
	Group C	-0.160	-0.370, 0.051	0.138
	First visit score	0.323	0.202, 0.445	1.8236E-7
Model 6 ( mean score in the fourth(W8) visit with considering first visit covariate)	Group D	-		
	Group A	-0.754	-1.031, -0.476	1.0022E-7
	Group B	-0.484	-0.857, -0.111	0.011
	Group C	-0.675	-0.925, -0.426	1.116E-7
	First visit score	0.471	0.326, 0.616	1.9497E-10

**Table 4:** Impact of symptoms on quality of life score based on NDI-10 scale

**Models**	**Parameter**	**β**	**95 % CI**	**p-value**
Model 1 (without considering any covariate)	Group D	-		
	Group A	-0.410	-0.708, -0.113	0.007
	Group B	0.069	-0.388, 0.525	0.768
	Group C	-0.289	-0.644, 0.065	0.110
Model 2 (considering first visit a covariate)	Group D	-		
	Group A	-0.282	-0.511, -0.053	0.016
	Group B	0.188	-0.181, 0.557	0.317
	Group C	-0.291	-0.585, 0.003	0.052
	First visit Score	0.282	0.187, 0.377	5.5706E-9
Model 3 (considering first visit, age and BMI covariates)	Group D	-		
	Group A	-0.272	-0.519, -0.025	0.031
	Group B	0.196	-0.180, 0.572	0.306
	Group C	-0.279	-0.592, 0.033	0.080
	First visit Score	0.275	0.182, 0.368	6.2063E-9
	Age	-0.002	-0.011, 0.008	0.709
	BMI	-0.007	-0.032, 0.018	0.590
Model 4 (mean score in the second (W2) visit)	Group D	-		
	Group A	0.034	-0.214, 0.282	0.786
	Group B	0.649	0.250, 1.049	0.001
	Group C	-0.051	-0.339, 0.237	0.727
	First visit score	0.317	0.220,0.413	1.3782E-10
Model 5 (mean score in the third (W4) visit considering first visit a covariate)	Group D	-		
	Group A	-0.119	-0.372, 0.134	0.357
	Group B	0.182	-0.244, 0.608	0.402
	Group C	-0.035	-0.384, 0.314	0.842
	First visit score	0.204	0.091, 0.316	3.85E-4
Model 6 ( mean score in the fourth(W8) visit with considering first visit a covariate)	Group D	-		
	Group A	-0.787	-1.189, -0.384	1.26E-4
	Group B	-0.480	-1.095, 0.134	0.126
	Group C	-0.807	-1.264, -0.350	0.001
	First visit score	0.315	0.177, 0.452	8E-6

**Table 5:** Mean and Standard deviation of total adjusted GSRS score

**Group**		**Before**	**Week 2**	**Week 4**	**Week 8**	**p-value**
Group A	Mean	48.08	28.96	22.96	24.44	<0.001**
	SD	12.490	7.977	8.692	8.889	
Group B	Mean	40.69	32.54	26.79	24.91	<0.001**
	SD	12.214	10.367	10.541	9.985	
Group C	Mean	40.73	25.04	21.62	22.13	<0.001**
	SD	15.351	5.250	5.643	5.766	
Group D	Mean	42.23	27.96	25.31	35.72	<0.001**
	SD	15.093	11.539	11.647	16.001	
p-value		0.156*	0.014*	0.342*	0.006*	

*Kruskal-Wallis Test, **Friedman Test

**Table 6:** Significant differences between omeprazole and other interventions in subscores and total scores of GSRS

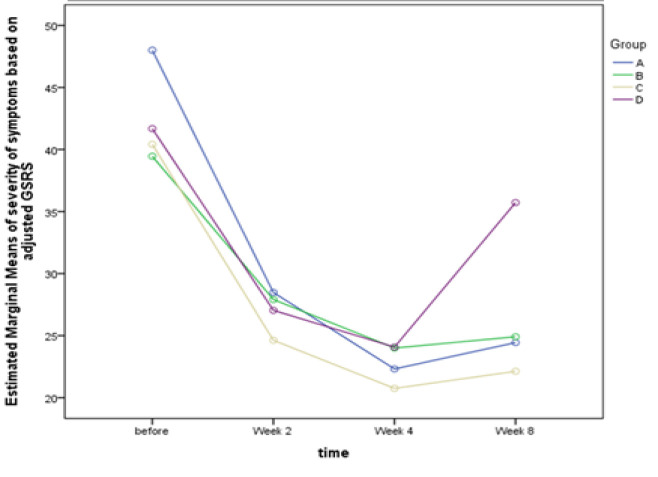

Models: model 1 analysis: Overall trend of the adjusted GSRS score and without considering any covariates, model 2 analysis: Overall trend of the adjusted GSRS score with considering first visit score as a covariate, model 3 analysis: Overall trend of the adjusted GSRS score with considering age and Body mass index (BMI) and first visit as covariates, model 4 analysis: The adjusted GSRS score with considering first visit score as a covariate in the second visit, model 5 analysis: The adjusted GSRS score with considering first visit score as a covariate in the third visit, model 6 analysis: The adjusted GSRS score with considering first visit score as a covariate in the Fourth visit. Group A: education on modification of eating and drinking habits and ‎sumac extract capsules, group B: education on modification of eating and drinking ‎habits, group C: sumac extract capsules, group D: ‎omeprazole capsules group.‎

**Table 7:** Significant differences between omeprazole and other interventions in subscores and total scores of NDI-10

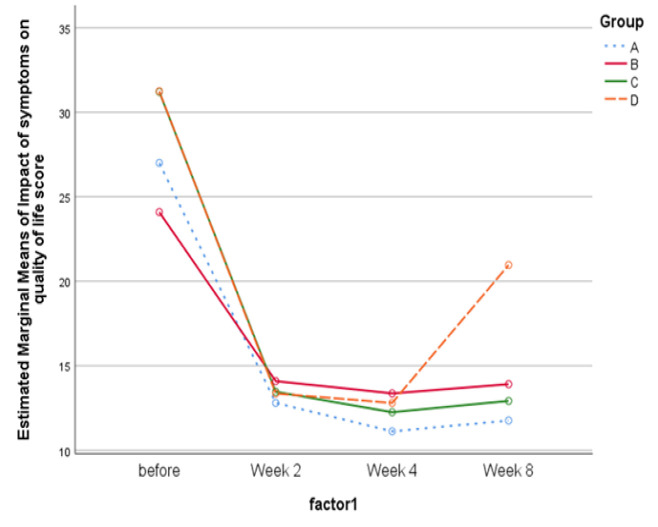

Models: model 1 analysis: Overall trend of the adjusted GSRS score and without considering any covariates, model 2 analysis: Overall trend of the adjusted GSRS score with considering first visit score as a covariate, model 3 analysis: Overall trend of the adjusted GSRS score with considering age and Body mass index (BMI) and first visit as covariates, model 4 analysis: The adjusted GSRS score with considering first visit score as a covariate in the second visit, model 5 analysis: The adjusted GSRS score with considering first visit score as a covariate in the third visit, model 6 analysis: The adjusted GSRS score with considering first visit score as a covariate in the Fourth visit. Group A: education on modification of eating and drinking habits and ‎sumac extract capsules, group B: education on modification of eating and drinking ‎habits, group C: sumac extract capsules, group D: ‎omeprazole capsules group.‎
